# High Sensitivity Surface Plasmon Resonance Sensor Based on Two-Dimensional MXene and Transition Metal Dichalcogenide: A Theoretical Study

**DOI:** 10.3390/nano9020165

**Published:** 2019-01-29

**Authors:** Yi Xu, Yee Sin Ang, Lin Wu, Lay Kee Ang

**Affiliations:** 1 SUTD-MIT International Design Center & Science and Math Cluster, Singapore University of Technology and Design (SUTD), 8 Somapah Road, Singapore 487372, Singapore; yi_xu@mymail.sutd.edu.sg (Y.X.); yeesin_ang@sutd.edu.sg (Y.S.A.); 2 Institute of High Performance Computing, Agency for Science, Technology, and Research (A*STAR), 1 Fusionopolis Way, #16-16 Connexis, Singapore 138632, Singapore; wul@ihpc.a-star.edu.sg

**Keywords:** MXene, Ti_3_C_2_T*_x_*, transition metal dichalcogenides, surface plasmon resonance, sensitivity

## Abstract

MXene, a new class of two-dimensional nanomaterials, have drawn increasing attention as emerging materials for sensing applications. However, MXene-based surface plasmon resonance sensors remain largely unexplored. In this work, we theoretically show that the sensitivity of the surface plasmon resonance sensor can be significantly enhanced by combining two-dimensional ${\mathrm{Ti}}_{3}C_{2}T_{x}$ MXene and transition metal dichalcogenides. A high sensitivity of $198^{\circ }$/RIU (refractive index unit) with a sensitivity enhancement of 41.43% was achieved in aqueous solutions (refractive index ∼1.33) with the employment of monolayer ${\mathrm{Ti}}_{3}C_{2}T_{x}$ MXene and five layers of ${\mathrm{WS}}_{2}$ at a 633 nm excitation wavelength. The integration of ${\mathrm{Ti}}_{3}C_{2}T_{x}$ MXene with a conventional surface plasmon resonance sensor provides a promising approach for bio- and chemical sensing, thus opening up new opportunities for highly sensitive surface plasmon resonance sensors using two-dimensional nanomaterials.

## 1. Introduction

Optical sensors based on surface plasmon resonance (SPR) has been widely used for biosensing and chemical sensing in the past few decades due to their superior characteristics, such as being highly sensitive, reliable, label-free, and their capacity for real-time detection [1,2,3,4,5]. Various types of SPR sensors [1,2], including prism-coupled SPR sensors, metallic-grating coupled SPR sensors, fiber optic SPR sensors, and waveguide-based SPR sensors, have been designed and demonstrated for sensing applications. The Kretschmann configuration [6] is a typical prism-coupled SPR sensor structure, in which plasmonic metal (e.g., gold) film is deposited onto the base of a prism. A transverse magnetic (TM)-polarized incident light undergoes total internal reflection at the prism/metal film interface and generates an evanescent wave that penetrates through the metal thin film. Thus exciting a surface plasmon at the interface between the metal film and sensing medium (i.e., the outer boundary of metal film). The excitation of the surface plasmons results in a resonant dip in the angular spectrum of the reflected light with a fixed excitation light wavelength. The excitation of the surface plasmon depends on the refractive index (RI) of the sensing medium (or analyte), and a slight change in the analyte RI will produce a variation in the position (i.e., resonance angle) and magnitude of the resonance dip. This variation of resonance angle can be employed for the sensitive detection of RI change [1,2].

To obtain a highly sensitive SPR sensor, various techniques have been proposed and demonstrated [7], such as coating a dielectric material on the metal film [8]. In recent years, graphene, a two-dimensional (2D) nanomaterial, has been proposed and implemented to improve the sensitivities of SPR sensors [9,10,11,12] due to its unusual optical properties [13,14,15,16,17,18,19]. For example, Wu et al. [9] first proposed a graphene-based SPR biosensor consising of a graphene-on-Au structure. This graphene-integrated SPR sensor exhibited enhanced sensitivity, compared to the bare Au-based conventional SPR sensor, and a sensitivity enhancement of 25% was achieved with 10 layers of graphene applied. Besides graphene, SPR sensors with 2D transition metal dichalcogenides (TMDs), including molybdenum disulfide (${\mathrm{MoS}}_{2}$), molybdenum diselenide (${\mathrm{MoSe}}_{2}$), tungsten disulfide (${\mathrm{WS}}_{2}$), and tungsten diselenide (${\mathrm{WSe}}_{2}$), have been studied [20,21,22,23,24,25]. Ouyang et al. [20] theoretically investigated the sensor performances of TMDs-based SPR sensors with the structure of Au/Si/TMDs under different excitation wavelengths. The highest RI sensitivity of 155.68${}^{\circ }/\mathrm{RIU}$ (RIU: refractive index unit) was obtained with the 35 nm Au/7 nm Si/monolayer ${\mathrm{WS}}_{2}$ structure at the wavelength of 600 nm. Another study on ${\mathrm{MoS}}_{2}$-integrated SPR sensors has demonstrated that the ${\mathrm{MoS}}_{2}$-based SPR sensor possesses better sensor performance (higher sensitivity and detection accuracy) than that of graphene-based sensors in the near-infrared regime [21].

MXenes [26,27,28], a new class of 2D materials consisting of transition metal carbides, nitrides, and carbonitrides, have attracted increasing attention in recent years due to their exceptional properties, including novel electrochemical properties [29] and extremely high electrical conductivity [30]. Furthermore, MXenes exhibit higly accessible hydrophilic surfaces [31], which is in contrast to graphene and most other 2D materials. Owing to their unique properties, MXenes have demonstrated promise for various applications, such as energy storage [31], water purification [32], chemical catalysts [33], photocatalysts [34], electrocatalysts [35], and photothermal therapy [36]. The MXene is also a promising material for sensing applications [37,38], such as electrochemical sensors [39,40], field effect transistor sensors [41], electrochemiluminescent sensors [42] and gas sensors [43,44]. For example, Kim et al. [44] recently demonstrated a ${\mathrm{Ti}}_{3}C_{2}T_{x}$ MXene gas sensor by making use of its high metallic conductivity and fully functionalized surface. This ${\mathrm{Ti}}_{3}C_{2}T_{x}$ MXene sensor exhibited higher sensitivity than that of gas sensors based on conventional semiconducting channel materials. It also possessed an ultra-high signal-to-noise ratio, which was two orders of magnitude greater than those of ${\mathrm{MoS}}_{2}$, black phosphorus, and reduced graphene oxide integrated sensors. Lorencova et al. [45] proposed and demonstrated a ${\mathrm{Ti}}_{3}C_{2}T_{x}$-based electrochemical sensor for $H_{2}O_{2}$ sensing. A detection limit of 0.7 nM was achieved, which is comparable to the best recorded so far (0.3 nM) [46]. However, few reports on MXene-integrated SPR sensors are available [47]. For example, a recent theoretical investigation on an ${\mathrm{Ti}}_{3}C_{2}T_{x}$ MXene-based SPR sensor [47] showed that coating ${\mathrm{Ti}}_{3}C_{2}T_{x}$ layers on Au film could enhance the sensitivity of a conventional Au-based SPR sensor. A RI sensitivity of 160${}^{\circ }/\mathrm{RIU}$ was achieved with four layers of ${\mathrm{Ti}}_{3}C_{2}T_{x}$-coated Au film at a 633 nm excitation wavelength, whereas it was 137${}^{\circ }/\mathrm{RIU}$ for the ${\mathrm{Ti}}_{3}C_{2}T_{x}$-devoid setup.

In this work, we designed a new MXene-based SPR sensor with the combination of ${\mathrm{Ti}}_{3}C_{2}T_{x}$ MXene and TMDs. The resulting structure exhibited significantly improved sensitivity compared to the 2D materials-devoid setup. A highest RI sensitivity of 198${}^{\circ }/\mathrm{RIU}$ was achieved for the Au/five-layer-${\mathrm{WS}}_{2}$/Au/monolayer ${\mathrm{Ti}}_{3}C_{2}T_{x}$ MXene structure in aqueous solutions with an excitation wavelength of 633 nm, which was a 41.43% sensitivity enhancement when compared with the conventional bare Au-based SPR sensor. The proposed MXene-TMDs plasmonic platform could offer new opportunities for highly sensitive SPR sensing. In addition, since the traditional prism-based SPR sensors have been successfully commericalized, such as Biacore (GE Healthcare), the proposed 2D nanomaterials-integrated SPR sensor could also stimulate new interest toward the exploration of commercially available high sensitivity SPR sensors.

## 2. Theoretical Model

**Figure 1:**
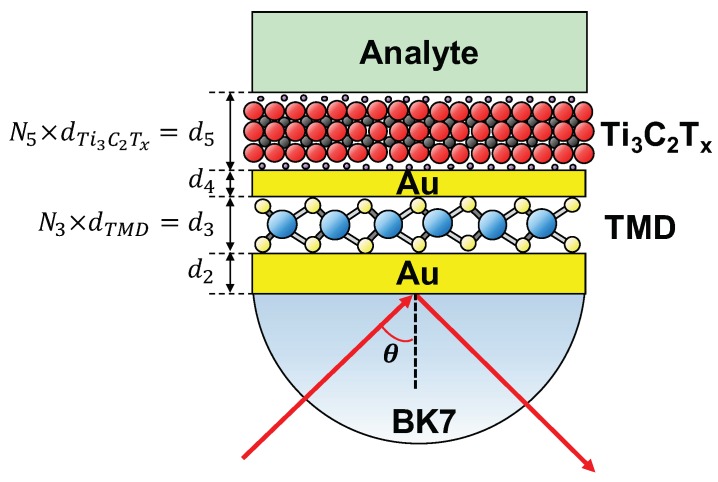
Schematic illustration of the proposed surface plasmon resonance (SPR) sensor with ${\mathrm{Ti}}_{3}C_{2}T_{x}$ and 2D transition metal dichalcogenides (TMD) layers.

The proposed SPR sensor structure is based on a modified Kretschmann configuration, as shown in Figure 1. In the proposed sensor structure, an Au film with the thickness of $d_{2}=50$ nm is attached to the base of a BK7 prism. Another thinner Au film ($d_{4}=10$ nm), decorated with TMDs and ${\mathrm{Ti}}_{3}C_{2}T_{x}$ MXene on each side, is deposited on the previous thick Au film (see Figure 1). The ${\mathrm{Ti}}_{3}C_{2}T_{x}$ MXene is kept in contact with the sensing medium or analyte. A TM-polarized light from a monochromatic source (λ=633 nm) is launched in one side of the BK7 prism and the reflected light is detected from the other side. By scanning the incident angle to obtain an angular spectrum of the reflected light, and monitoring the resonance angle shift, the analyte RI variations can be observed.

The reflectance *R* of the proposed sensor can be calculated with a generalized N-layer model [48]. The reflectance for the TM-polarized incident light is:

(1) $$R={\left|\frac{\left(M_{11}+M_{12}q_{N}\right)q_{1}-\left(M_{21}+M_{22}q_{N}\right)}{\left(M_{11}+M_{12}q_{N}\right)q_{1}+\left(M_{21}+M_{22}q_{N}\right)}\right|}^{2},$$

in which $M_{11}$, $M_{12}$, $M_{21}$, and $M_{22}$ are the four elements of the matrix *M* given by:

(2) $$M=\left[\begin{array}{cc} M_{11} & M_{12}\\ M_{21} & M_{22} \end{array}\right]=\prod_{k=2}^{N-1}M_{k},$$

with:

(3) $$M_{k}=\left[\begin{array}{cc} \cos\beta_{k} & -i\left(\sin\beta_{k}\right)/q_{k}\\ -iq_{k}\sin\beta_{k} & \cos\beta_{k} \end{array}\right].$$

Here,

(4) $$\beta_{k}=\frac{2\pi d_{k}}{\lambda }{\left(n_{k}^{2}-n_{1}^{2}\sin^{2}\theta_{1}\right)}^{1/2},$$

and

(5) $$q_{k}=\frac{{\left(n_{k}^{2}-n_{1}^{2}\sin^{2}\theta_{1}\right)}^{1/2}}{n_{k}^{2}},$$

in which λ is the wavelength of incident TM-polarized light, and $\theta_{1}$ is the incident angle. $d_{k}$ and $n_{k}$ are the thickness and RI of the *k*th layer with k=2 to N−1, respectively. The first layer (k=1) in the sensor structure is the BK7 prism, and the wavelength-dependent RI is given by [49]:

(6) $$n_{\mathrm{BK}7}=\sqrt{1+\frac{1.03961212\lambda^{2}}{\lambda^{2}-0.00600069867}+\frac{0.231792344\lambda^{2}}{\lambda^{2}-0.0200179144}+\frac{1.01046945\lambda^{2}}{\lambda^{2}-103.560653}},$$

in which the wavelength λ is given in μm. The *N*th layer is the analyte, and its RI is defined as $n_{a}=1.33$ (water). The complex RI of Au film is calculated according to the Drude–Lorentz model [50]:

(7) $$n_{\mathrm{Au}}=\sqrt{1-\frac{\lambda^{2}\lambda_{c}}{\lambda_{p}^{2}\left(\lambda_{c}+i\lambda \right)}},$$

where $\lambda_{c}$ (=$8.9342\times 10^{-6}$ m) and $\lambda_{p}$ (=$1.6826\times 10^{-7}$ m) is the collision wavelength and the plasma wavelength of Au, respectively. Monolayer ${\mathrm{Ti}}_{3}C_{2}T_{x}$ has a thickness of $d_{{\mathrm{Ti}}_{3}C_{2}T_{x}}=0.993$ nm [51], and its refractive index is 2.38+1.33i at the wavelength of 633 nm [52]. For monolayer TMD, the thickness is 0.65 nm, 0.7 nm, 0.8 nm and 0.7 nm for ${\mathrm{MoS}}_{2}$, ${\mathrm{MoSe}}_{2}$, ${\mathrm{WS}}_{2}$ and ${\mathrm{WSe}}_{2}$, respectively. And the corresponding complex RI at the wavelength of 633 nm is 5.0805+1.1723i, 4.6226+1.0063i, 4.8937+0.3124i, and 4.5501+0.4332i, respectively [23,53,54]. In the proposed sensor structure, the layer number of the TMD is $N_{3}$, and it is $N_{5}$ for ${\mathrm{Ti}}_{3}C_{2}T_{x}$. The reflectance *R* depends on the analyte RI $n_{a}$, and a variation of analyte RI $\Delta n_{a}$ will result in a change in the reflectance, as well as the resonance angle $\Delta \theta_{res}$. Therefore, the sensitivity is defined as:

(8) $$S=\frac{\Delta \theta_{res}}{\Delta n_{a}}.$$

## 3. Results and Discussion

**Figure 2:**
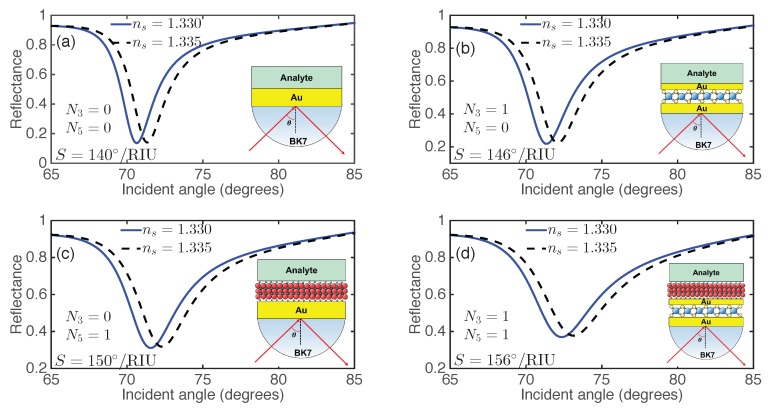
Reflectance as a function of the incident angle before (solid lines) and after (dashed lines) the variation of analyte refractive index (RI) for the ${\mathrm{Ti}}_{3}C_{2}T_{x}$-${\mathrm{MoS}}_{2}$-based SPR sensor with (**a**) $N_{3}=0$, $N_{5}=0$, (i.e., no 2D materials); (**b**) $N_{3}=1$, $N_{5}=0$, (i.e., monolayer ${\mathrm{MoS}}_{2}$); (**c**) $N_{3}=0$, $N_{5}=1$, (i.e., monolayer ${\mathrm{Ti}}_{3}C_{2}T_{x}$), and (**d**) $N_{3}=1$, $N_{5}=1$, (i.e., monolayer ${\mathrm{MoS}}_{2}$ and monolayer ${\mathrm{Ti}}_{3}C_{2}T_{x}$).

2D material-on-Au has been experimentally obtained in recent years. For example, graphene on Au surface has been experimentally demonstrated using the transfer printing technique [55,56]. The obtained graphene-on-Au structure was experimentally demonstrated for SPR sensing applications [56]. TMDs on the Au surface were also experimentally achieved [57,58,59,60,61,62]. These techniques can be applied for the fabrication of MXene-on-Au structures. Therefore, the proposed SPR sensor based on 2D MXene and TMDs are expected to be achieved easily. In order to illustrate the sensitivity enhancement of the proposed SPR sensor, we calculated the angular spectrum of the reflected light for various sensor structures, as shown in Figure 2, before (solid lines) and after (dashed lines) the RI variation of the sensing medium, assuming a small RI change $\Delta n_{a}=0.005$. For each SPR sensor, the increase of the analyte RI will shift the resonance angle toward a larger value. For example, for the SPR sensor with $N_{3}=0$ and $N_{5}=0$ (i.e., conventional SPR sensor with 60 nm (=$d_{2}+d_{4}$) Au film shown in Figure 2a), the resonance angle is $70.64{}^{\circ }$ with the ambient RI of 1.330, and increases to $71.34{}^{\circ }$ with a small analyte RI increment ($\Delta n_{a}=0.005$). Therefore, a sensitivity of $S_{0}=140^{\circ }/\mathrm{RIU}$ was obtained for the bare Au-based SPR sensor. By inserting a monolayer ${\mathrm{MoS}}_{2}$ between the two Au films (i.e., $N_{3}=1$ and $N_{5}=0$), an enhanced sensitivity of $S=146^{\circ }/\mathrm{RIU}$ was achieved (see Figure 2b). To study the sensitivity improvement with reference to the sensitivity of the conventional Au-based SPR sensor, we denoted the sensitivity enhancement as $\left(S-S_{0}\right)/S_{0}\times 100\%$, in which *S* is the sensitivity of 2D-nanomaterial-integrated SPR sensor. For the SPR sensor shown in Figure 2b, a relatively low sensitivity enhancement of 4.29% was obtained. The sensitivity and sensitivity enhancement were improved to $150^{\circ }/\mathrm{RIU}$ and 7.14%, respectively, with only one layer of ${\mathrm{Ti}}_{3}C_{2}T_{x}$ (i.e., $N_{3}=0$, $N_{5}=1$, Figure 2c). With the employment of both a ${\mathrm{Ti}}_{3}C_{2}T_{x}$ MXene and ${\mathrm{MoS}}_{2}$ layer ($N_{3}=1$ and $N_{5}=1$), an enhanced sensitivity of $S=156^{\circ }/\mathrm{RIU}$ with the sensitivity enhancement of 11.43% was achieved, as shown in Figure 2d. Besides the ${\mathrm{Ti}}_{3}C_{2}T_{x}$-${\mathrm{MoS}}_{2}$-based SPR sensor, three other TMDs (${\mathrm{MoSe}}_{2}$, ${\mathrm{WS}}_{2}$, ${\mathrm{WSe}}_{2}$) and ${\mathrm{Ti}}_{3}C_{2}T_{x}$ integrated SPR sensors ($N_{3}=1$ and $N_{5}=1$) also exhibited enhanced sensitivity (Figures S1–S3 in the Supporting Information). Therefore, the proposed SPR sensor with the simultaneous employment of ${\mathrm{Ti}}_{3}C_{2}T_{x}$ and TMDs exhibited enhanced sensitivity and offers the potential for highly sensitive sensing applications.

**Figure 3:**
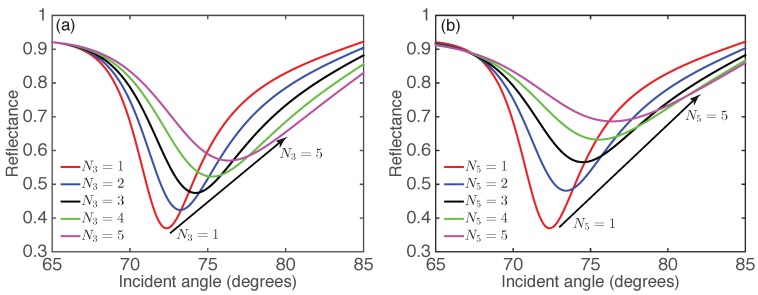
Reflectance as a function of the incident angle for ${\mathrm{Ti}}_{3}C_{2}T_{x}$-${\mathrm{MoS}}_{2}$-based SPR sensor with (**a**) different number of ${\mathrm{MoS}}_{2}$ ($N_{3}$) and monolayer ${\mathrm{Ti}}_{3}C_{2}T_{x}$ ($N_{5}=1$), and (**b**) different number of ${\mathrm{Ti}}_{3}C_{2}T_{x}$ ($N_{5}$) and monolayer ${\mathrm{MoS}}_{2}$ ($N_{3}=1$).

The study above only focuses on monolayer ${\mathrm{MoS}}_{2}$ and ${\mathrm{Ti}}_{3}C_{2}T_{x}$. Previous investigations on 2D-material-integrated SPR sensors have demonstrated that the sensitivity also depends on the layer number of 2D materials [9,10,11,20,21,22,23,24]. Therefore, it is necessary to study the effect of number of ${\mathrm{Ti}}_{3}C_{2}T_{x}$ and ${\mathrm{MoS}}_{2}$ layers on the sensitivity. First, we investigated the effect of multiple layers of 2D materials on the reflectance for the proposed SPR sensor. The reflectance as a function of the incident angle for the monolayer ${\mathrm{Ti}}_{3}C_{2}T_{x}$-${\mathrm{MoS}}_{2}$-based SPR sensor with different numbers of ${\mathrm{MoS}}_{2}$ layers is shown in Figure 3a. It was readily apparent that the resonance angle increased with the number of ${\mathrm{MoS}}_{2}$ layers due to the increased propagation constant (wavector) of the surface plasmons. In addition, a shallowing and broadening of the reflectance curves was observed when the layers of ${\mathrm{MoS}}_{2}$ increased, due to the increased electron energy loss [20,22]. Similar phenomena were found in the reflectance curves for ${\mathrm{Ti}}_{3}C_{2}T_{x}$-monolayer ${\mathrm{MoS}}_{2}$-based SPR sensors with different numbers of ${\mathrm{Ti}}_{3}C_{2}T_{x}$ layers, as shown in Figure 3b. By comparing Figure 3a and Figure 3b, it was found that the increased energy loss caused by the integration of ${\mathrm{Ti}}_{3}C_{2}T_{x}$ layers was larger than that caused by the additional ${\mathrm{MoS}}_{2}$ layers.

**Figure 4:**
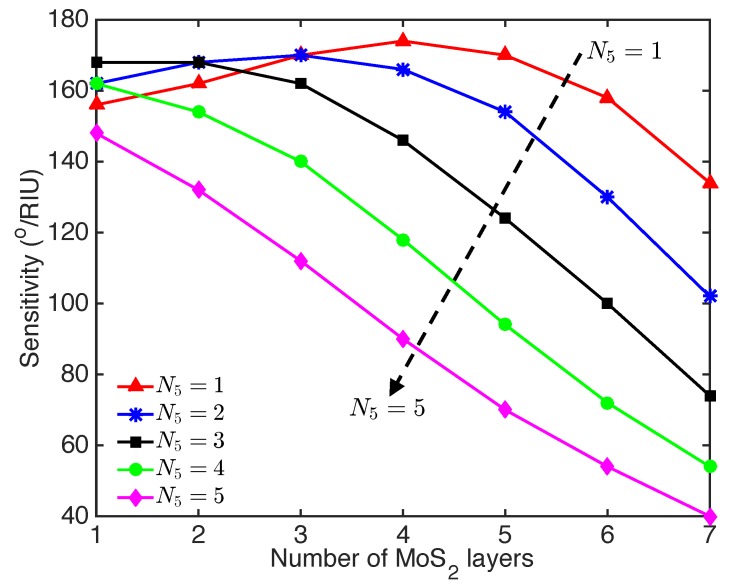
Sensitivity as a function of the number of ${\mathrm{MoS}}_{2}$ layers for ${\mathrm{Ti}}_{3}C_{2}T_{x}$-${\mathrm{MoS}}_{2}$-based SPR sensor with different layers of ${\mathrm{Ti}}_{3}C_{2}T_{x}$.

To further improve the sensitivity of proposed SPR sensor, we studied the optimiziation of the sensitivity by varying the layer number of the ${\mathrm{Ti}}_{3}C_{2}T_{x}$ MXene and TMDs. The sensitivity as a function of the number of ${\mathrm{MoS}}_{2}$ layers for the ${\mathrm{Ti}}_{3}C_{2}T_{x}$-${\mathrm{MoS}}_{2}$-based SPR sensor with different numbers of ${\mathrm{Ti}}_{3}C_{2}T_{x}$ layers is shown in Figure 4. The sensitivity first increased and then decreased with the number of ${\mathrm{MoS}}_{2}$ layers, when the SPR sensor integrated monolayer and two layers of ${\mathrm{Ti}}_{3}C_{2}T_{x}$. However, adding more layers of ${\mathrm{Ti}}_{3}C_{2}T_{x}$ (e.g., three to five layers) resulted in decreased sensitivity with the number of ${\mathrm{MoS}}_{2}$ layers. Due to the relative higher energy loss of the ${\mathrm{Ti}}_{3}C_{2}T_{x}$ layers, the SPR signal enhancement effect of the ${\mathrm{MoS}}_{2}$ layers in the SPR sensor with three to five layers of ${\mathrm{Ti}}_{3}C_{2}T_{x}$ was overwhelmed by the energy loss with the additional ${\mathrm{MoS}}_{2}$ layers. In contrast, with the integration of monolayer ${\mathrm{Ti}}_{3}C_{2}T_{x}$, the sensitivity increased with the number of ${\mathrm{MoS}}_{2}$ layers from one to four (see Figure 4), where the SPR signal enhancement effect was more significant than the energy loss caused by the ${\mathrm{MoS}}_{2}$ layers [22]. The maximum sensitivity of $174^{\circ }/\mathrm{RIU}$ was found for the ${\mathrm{Ti}}_{3}C_{2}T_{x}$-${\mathrm{MoS}}_{2}$-based SPR sensor integrated with four-layer ${\mathrm{MoS}}_{2}$ and monolayer ${\mathrm{Ti}}_{3}C_{2}T_{x}$.

The optimization of various combinations of ${\mathrm{Ti}}_{3}C_{2}T_{x}$ MXene and TMDs (e.g., ${\mathrm{Ti}}_{3}C_{2}T_{x}$-${\mathrm{MoSe}}_{2}$, ${\mathrm{Ti}}_{3}C_{2}T_{x}$-${\mathrm{WS}}_{2}$, and ${\mathrm{Ti}}_{3}C_{2}T_{x}$-${\mathrm{WSe}}_{2}$) of the SPR sensors are shown in Figures S4–S6 of the Supporting Information. It was found that only monolayer ${\mathrm{Ti}}_{3}C_{2}T_{x}$ MXene could be used to obtain the maximum sensitivity for the ${\mathrm{Ti}}_{3}C_{2}T_{x}$-TMDs-based SPR sensors. The sensitivity and sensitivity enhancement at the optimized number of TMD layers and ${\mathrm{Ti}}_{3}C_{2}T_{x}$ MXene layers for the proposed SPR sensor structure are summarized in Table 1. The ${\mathrm{Ti}}_{3}C_{2}T_{x}$-${\mathrm{WS}}_{2}$- and ${\mathrm{Ti}}_{3}C_{2}T_{x}$-${\mathrm{WSe}}_{2}$-based SPR sensors possessed sensitivities more than $190^{\circ }/\mathrm{RIU}$. A maximum sensitivity of $198^{\circ }/\mathrm{RIU}$ was achieved with the sensor structure of Au/${\mathrm{WSe}}_{2}$ (six layers)/${\mathrm{Ti}}_{3}C_{2}T_{x}$ (one layer)/Au, and a sensitivity enhancement of 41.43% was obtained. The sensitivities achieved with the proposed ${\mathrm{Ti}}_{3}C_{2}T_{x}$-TMDs-based SPR sensors at a 633 nm excitation wavelength were significantly higher than that of the conventional Au-${\mathrm{Ti}}_{3}C_{2}T_{x}$ (four layer)-based SPR sensor ($160^{\circ }/\mathrm{RIU}$) recently reported by Wu et al. [47]. The combination of TMDs and ${\mathrm{Ti}}_{3}C_{2}T_{x}$ offers the alternative of sensitivity enhancement for ${\mathrm{Ti}}_{3}C_{2}T_{x}$-based SPR sensors.

**Figure 5:**
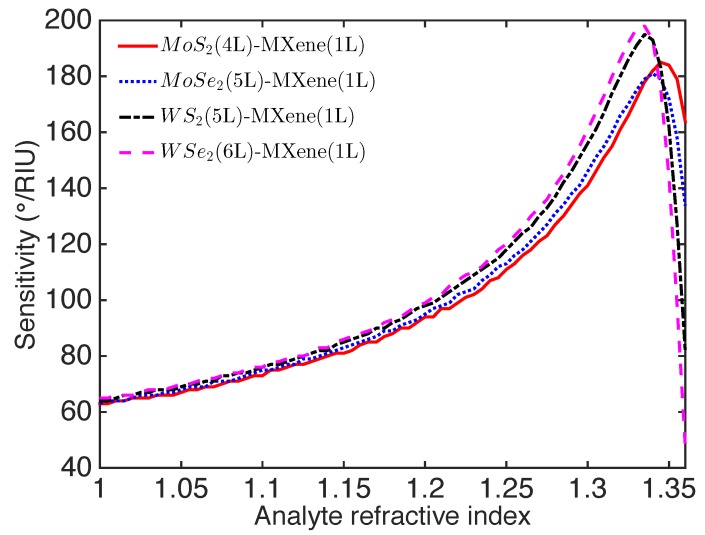
Variation of sensitivity for the optimized ${\mathrm{Ti}}_{3}C_{2}T_{x}$-TMD-based SPR sensor with the varying analyte RI.

The RI of the surrounding environment was also important to the sensitivity, which determined the appropriate working RI range or working environment (e.g., gas or liquid) of the SPR sensor. The sensitivity for the optimized ${\mathrm{Ti}}_{3}C_{2}T_{x}$-TMDs-based SPR sensor was plotted with varying analyte RI in Figure 5. The optimized ${\mathrm{Ti}}_{3}C_{2}T_{x}$-TMDs-based SPR sensor possessed a relatively low sensitivity (<90${}^{\circ }/\mathrm{RIU}$) within the analyte RI range from 1.0 to 1.15. This revealed that the proposed SPR sensor was not appropriate for gas sensing, which typically involves a RI ∼1.0. The sensitivity of the optimized SPR sensor first increased to a maximum and then decreased with the analyte RI in the range of 1.0–1.36. The maximum RI sensitivity was found around the analyte RI of 1.330 (i.e., the RI of water). Therefore, the proposed sensor was more suited for operating in an aqueous medium, particularly for bio- and chemical sensing.

## 4. Conclusions

A novel SPR sensor based on Au-${\mathrm{Ti}}_{3}C_{2}T_{x}$-Au-TMDs is theoretically presented. The MXene-TMDs-integrated SPR sensor possessed enhanced sensitivity as compared to the bare Au film-based SPR sensor. For the aqueous solutions (RI ∼1.33), the RI sensitivities of $174^{\circ }/\mathrm{RIU}$, $176^{\circ }/\mathrm{RIU}$, $198^{\circ }/\mathrm{RIU}$, and $192^{\circ }/\mathrm{RIU}$ for the proposed SPR sensor with monolayer ${\mathrm{Ti}}_{3}C_{2}T_{x}$ MXene and four-layer ${\mathrm{MoS}}_{2}$, five-layer ${\mathrm{MoSe}}_{2}$, five-layer ${\mathrm{WS}}_{2}$, and six-layer ${\mathrm{WSe}}_{2}$, respectively, were achieved at the 633 nm excitation wavelength. Compared to the conventional Au film SPR sensor, the sensitivity was significantly enhanced by 24.29%, 25.71%, 41.43%, and 37.14%, respectively. The high sensitivities of the proposed ${\mathrm{Ti}}_{3}C_{2}T_{x}$ MXene-based SPR sensors offer a potential route towards highly sensitive SPR sensors. Although this work was purely based on theoretical calculations, we used realistic material parameters and the results could be readily verified by experimental investigations. Moreover, since the structures of graphene-on-Au and TMDs-on-Au have been experimentally realized in recent years [55,56,57,58,59,60,61,62], it is possible to fabricate the MXene-on-Au structure. Thus the proposed SPR sensor based on 2D MXene and TMDs is experimentally feasible.

## Figures and Tables

**Table 1:** Sensitivity and sensitivity enhancement at the optimized number of TMD layers and ${\mathrm{Ti}}_{3}C_{2}T_{x}$ layers for the ${\mathrm{Ti}}_{3}C_{2}T_{x}$-TMDs-based SPR sensor.

Type of TMD	Number of TMD Layers *N*_3_	Number of ${\mathrm{Ti}}_{3}C_{2}T_{x}$ Layers *N*_5_	Sensitivity (°/RIU)	$\left(S-S_{0}\right)/S_{0}$ (%)
${\mathrm{MoS}}_{2}$	4	1	174	24.29
${\mathrm{MoSe}}_{2}$	5	1	176	25.71
${\mathrm{WS}}_{2}$	5	1	198	41.43
${\mathrm{WSe}}_{2}$	6	1	192	37.14

## Supplementary Materials

The following are available online at http://www.mdpi.com/2079-4991/9/2/165/s1, Figure S1: Reflectance for the ${\mathrm{Ti}}_{3}C_{2}T_{x}$-${\mathrm{MoSe}}_{2}$-based SPR sensor; Figure S2: Reflectance for the ${\mathrm{Ti}}_{3}C_{2}T_{x}$-${\mathrm{WS}}_{2}$-based SPR sensor; Figure S3: Reflectance for the ${\mathrm{Ti}}_{3}C_{2}T_{x}$-${\mathrm{WSe}}_{2}$-based SPR sensor; Figure S4: Variation of sensitivity with number of ${\mathrm{MoSe}}_{2}$ and ${\mathrm{Ti}}_{3}C_{2}T_{x}$ layers for the ${\mathrm{Ti}}_{3}C_{2}T_{x}$-${\mathrm{MoSe}}_{2}$-based SPR sensor; Figure S5: Variation of sensitivity with number of ${\mathrm{WS}}_{2}$ and ${\mathrm{Ti}}_{3}C_{2}T_{x}$ layers for the ${\mathrm{Ti}}_{3}C_{2}T_{x}$-${\mathrm{WS}}_{2}$-based SPR sensor; Figure S6: Variation of sensitivity with number of ${\mathrm{WSe}}_{2}$ and ${\mathrm{Ti}}_{3}C_{2}T_{x}$ layers for the ${\mathrm{Ti}}_{3}C_{2}T_{x}$-${\mathrm{WSe}}_{2}$-based SPR sensor.
