# Supplementary material for: High Sensitivity Surface Plasmon Resonance Sensor Based on Two-Dimensional MXene and Transition Metal Dichalcogenide: A Theoretical Study
Source: Nanomaterials (Basel). 2019 Jan 29;9(2):165. doi: 10.3390/nano9020165 (PMC6409766; doi:10.3390/nano9020165)
Supplement: Supplementary file 1 [file nanomaterials-09-00165-s001.zip › supporting_information_revised/supporting_information.pdf]

Supporting Information

Two-dimensional transition metal dichalcogenides mediated long range surface plasmon resonance biosensors

Yi Xu,[1,2] Yee Sin Ang,[1] Lin Wu,[2] and L. K. Ang[1,*]

[1] *SUTD-MIT International Design Center & Science and Math Cluster, Singapore University of Technology and Design (SUTD), 8 Somapah Road, Singapore 487372*

[2] *Institute of High Performance Computing, Agency for Science, Technology, and Research (A*STAR), 1 Fusionopolis Way, #16-16 Connexis, Singapore 138632*

[*] ricky_ang@sutd.edu.sg

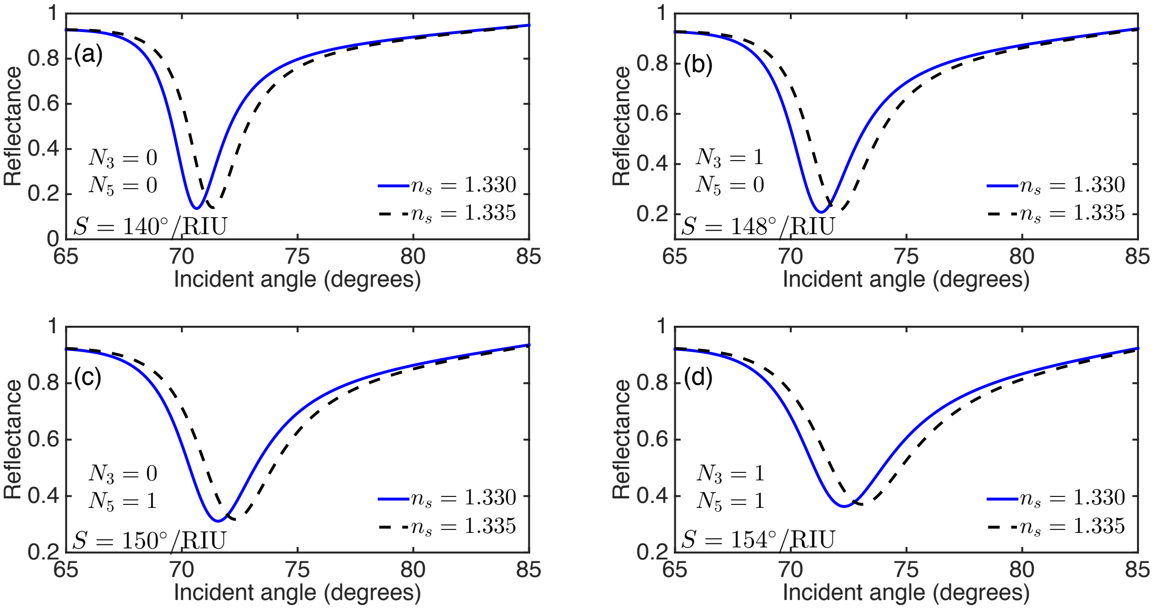

Figure S1. Reflectance as a function of the incident angle before (solid lines) and after (dashed lines) the variation of analyte RI for the $Ti_3C_2T_x$-$MoSe_2$-based SPR sensor with (**a**) $N_3 = 0$, $N_5 = 0$, (**b**) $N_3 = 1$, $N_5 = 0$, (**c**) $N_3 = 0$, $N_5 = 1$, and (**d**) $N_3 = 1$, $N_5 = 1$.

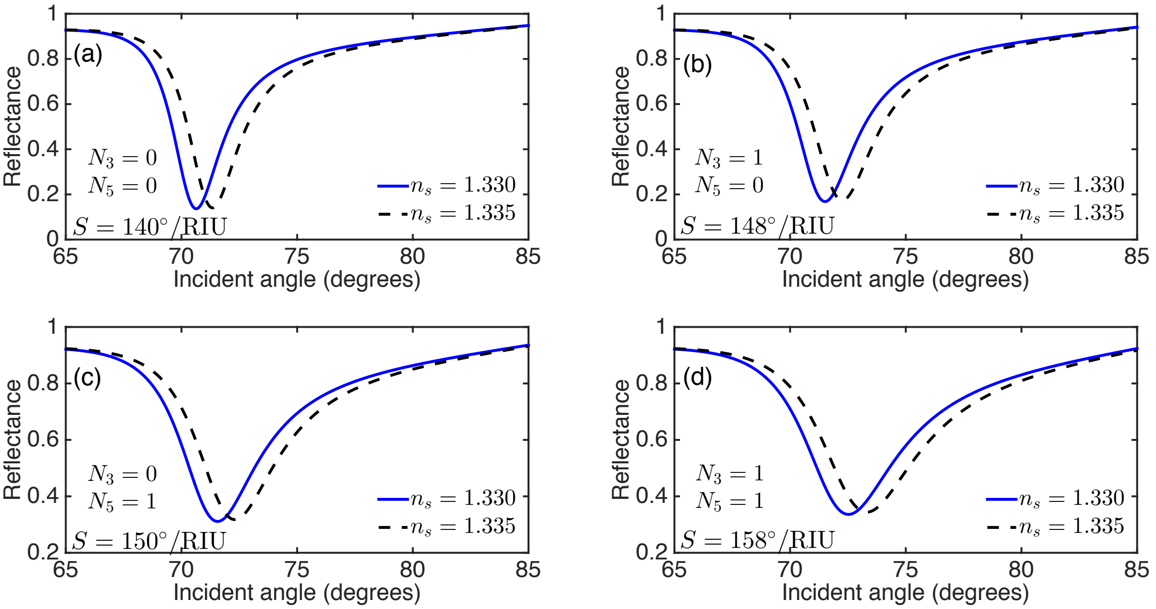

Figure S2. Reflectance as a function of the incident angle before (solid lines) and after (dashed lines) the variation of analyte RI for the $Ti_3C_2T_x$-WS_2-based SPR sensor with (**a**) $N_3 = 0$, $N_5 = 0$, (**b**) $N_3 = 1$, $N_5 = 0$, (**c**) $N_3 = 0$, $N_5 = 1$, and (**d**) $N_3 = 1$, $N_5 = 1$.

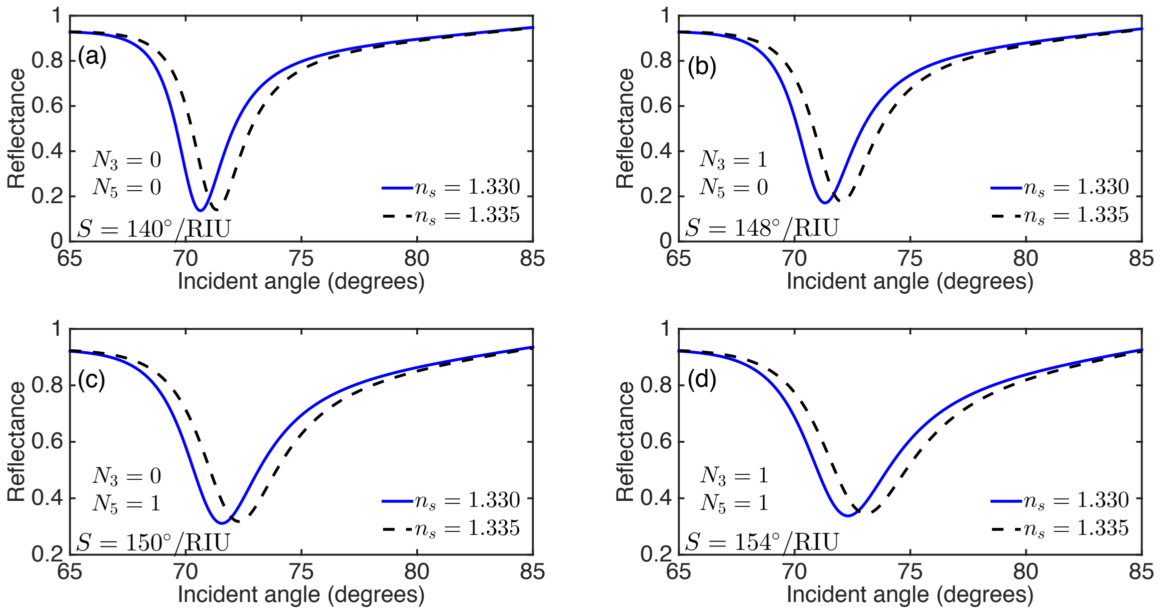

Figure S3. Reflectance as a function of the incident angle before (solid lines) and after (dashed lines) the variation of analyte RI for the $Ti_3C_2T_x$-WSe_2-based SPR sensor with (**a**) $N_3 = 0$, $N_5 = 0$, (**b**) $N_3 = 1$, $N_5 = 0$, (**c**) $N_3 = 0$, $N_5 = 1$, and (**d**) $N_3 = 1$, $N_5 = 1$.

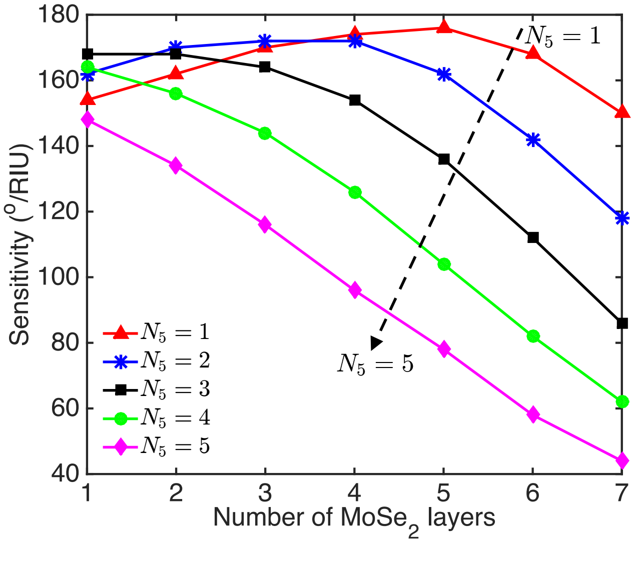

Figure S4. Sensitivity as a function of the number of $MoSe_2$ layers for $Ti_3C_2T_x$-$MoSe_2$-based SPR sensor with different layers of $Ti_3C_2T_x$.

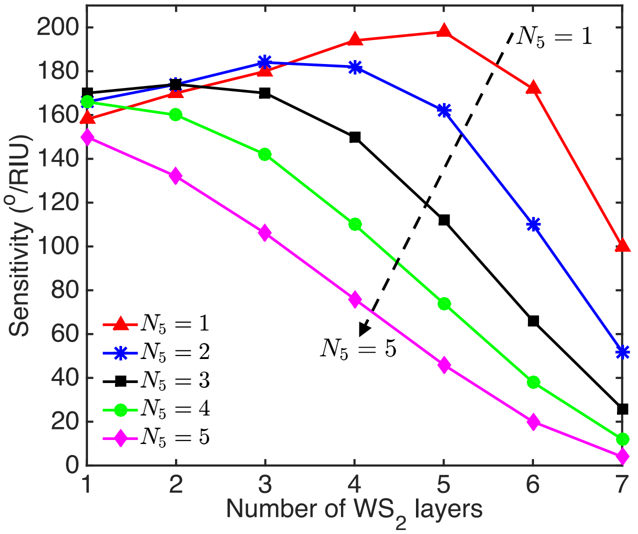

Figure S5. Sensitivity as a function of the number of WS_2 layers for $Ti_3C_2T_x$-WS_2-based SPR sensor with different layers of $Ti_3C_2T_x$.

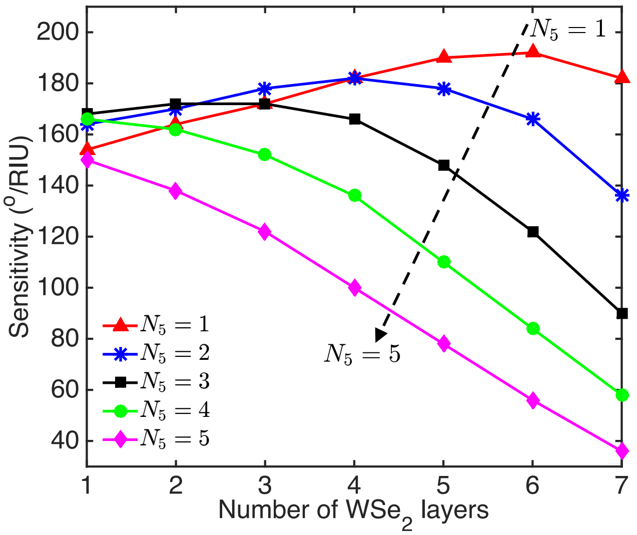

Figure S6. Sensitivity as a function of the number of WSe_2 layers for $Ti_3C_2T_x$-WSe_2-based SPR sensor with different layers of $Ti_3C_2T_x$.
