# Supplementary figures and images for: High Sensitivity Surface Plasmon Resonance Sensor Based on Two-Dimensional MXene and Transition Metal Dichalcogenide: A Theoretical Study
Source: Nanomaterials (Basel). 2019 Jan 29;9(2):165. doi: 10.3390/nano9020165 (PMC6409766; doi:10.3390/nano9020165)

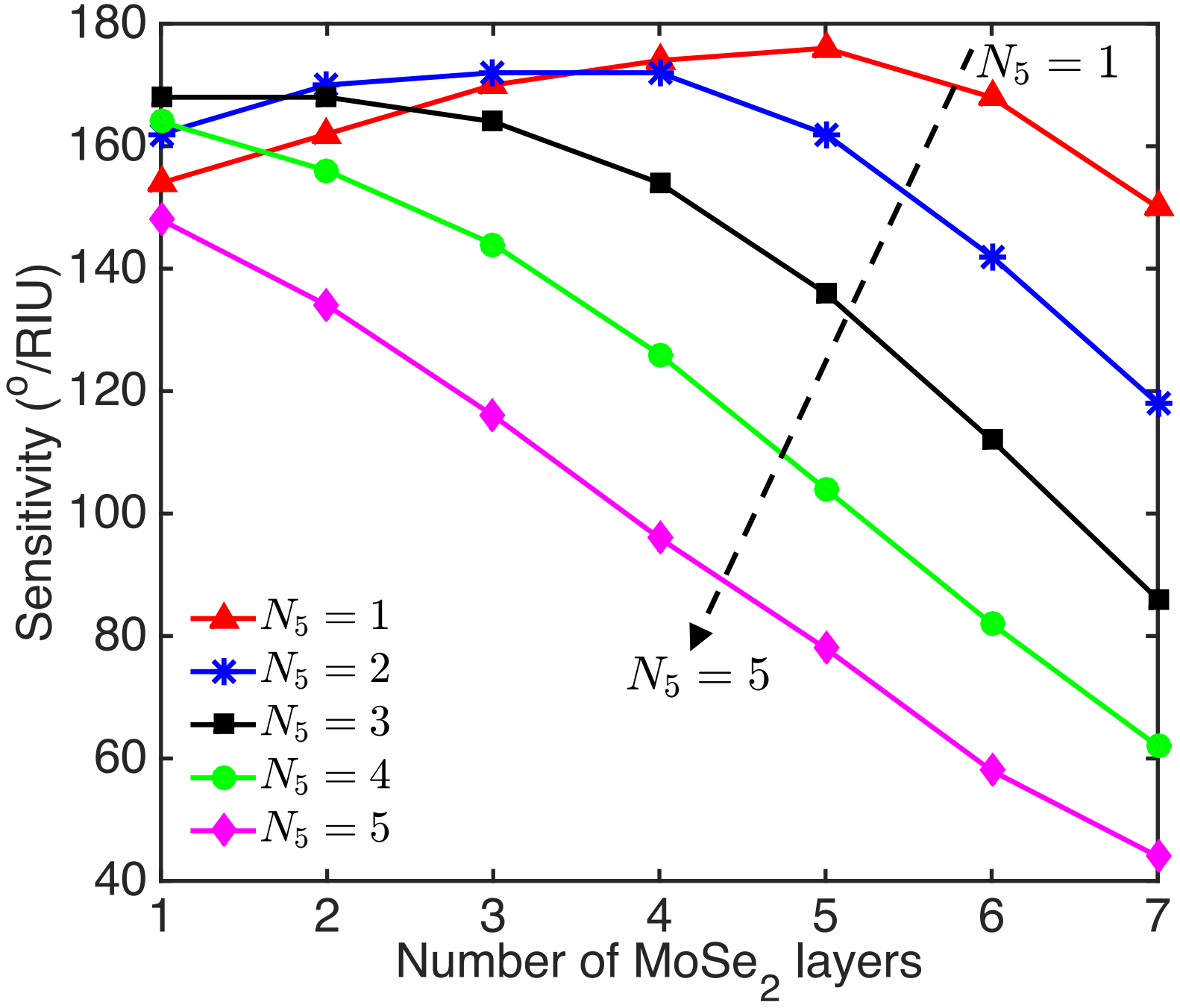

Supplement: Supplementary file 1 [file nanomaterials-09-00165-s001.zip › supporting_information_revised/figs4.pdf]

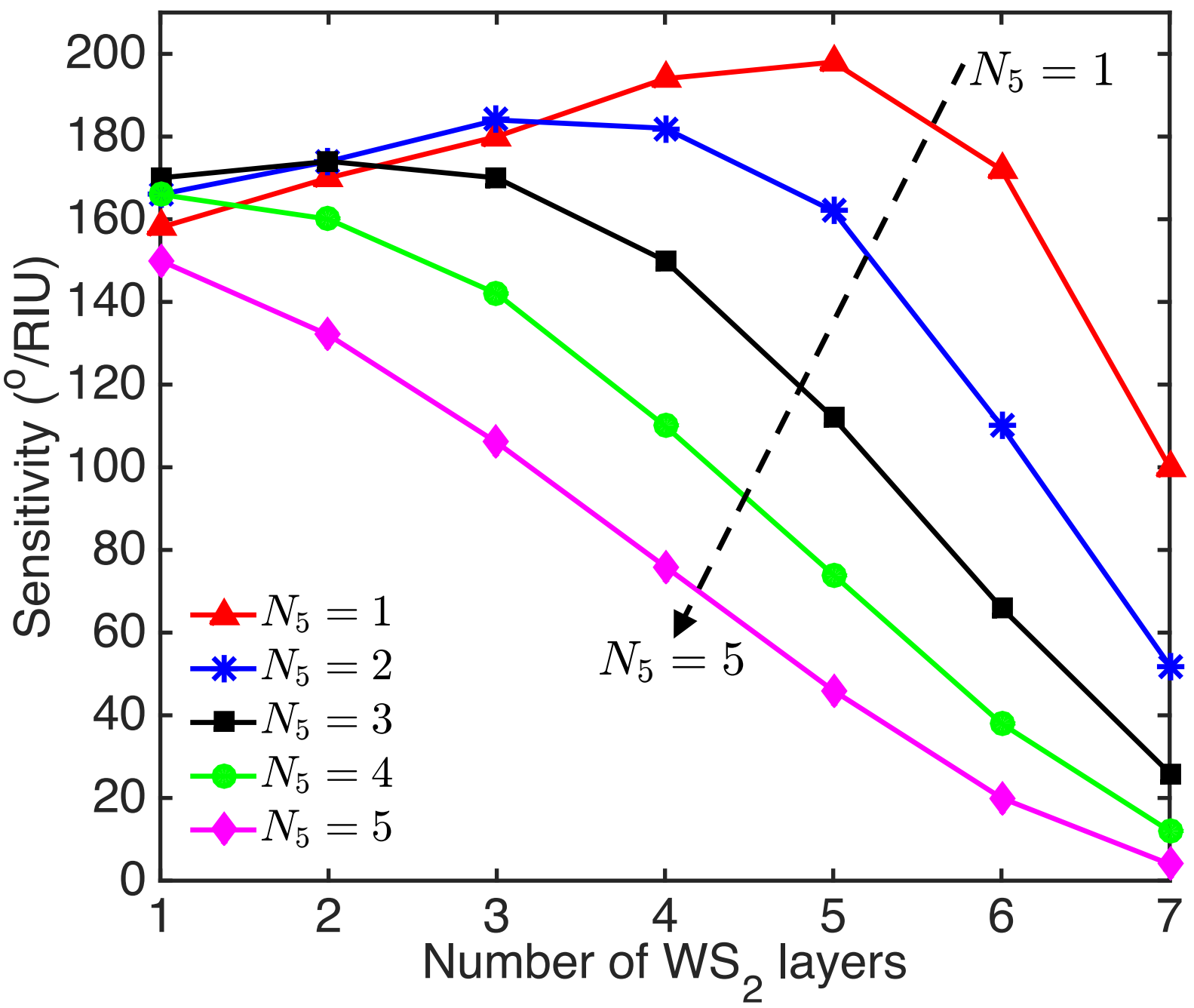

Supplement: Supplementary file 1 [file nanomaterials-09-00165-s001.zip › supporting_information_revised/figs5.pdf]

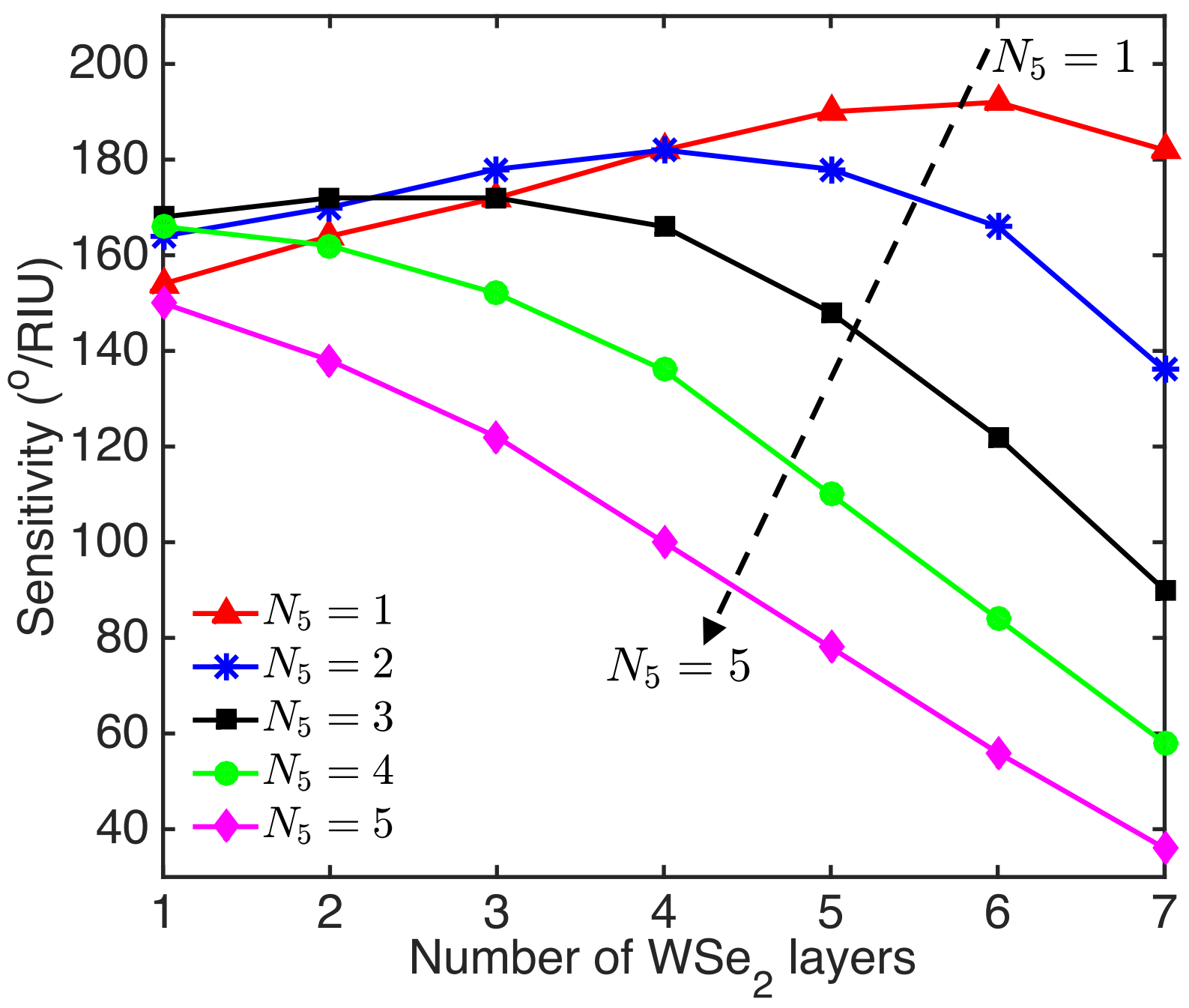

Supplement: Supplementary file 1 [file nanomaterials-09-00165-s001.zip › supporting_information_revised/figs6.pdf]

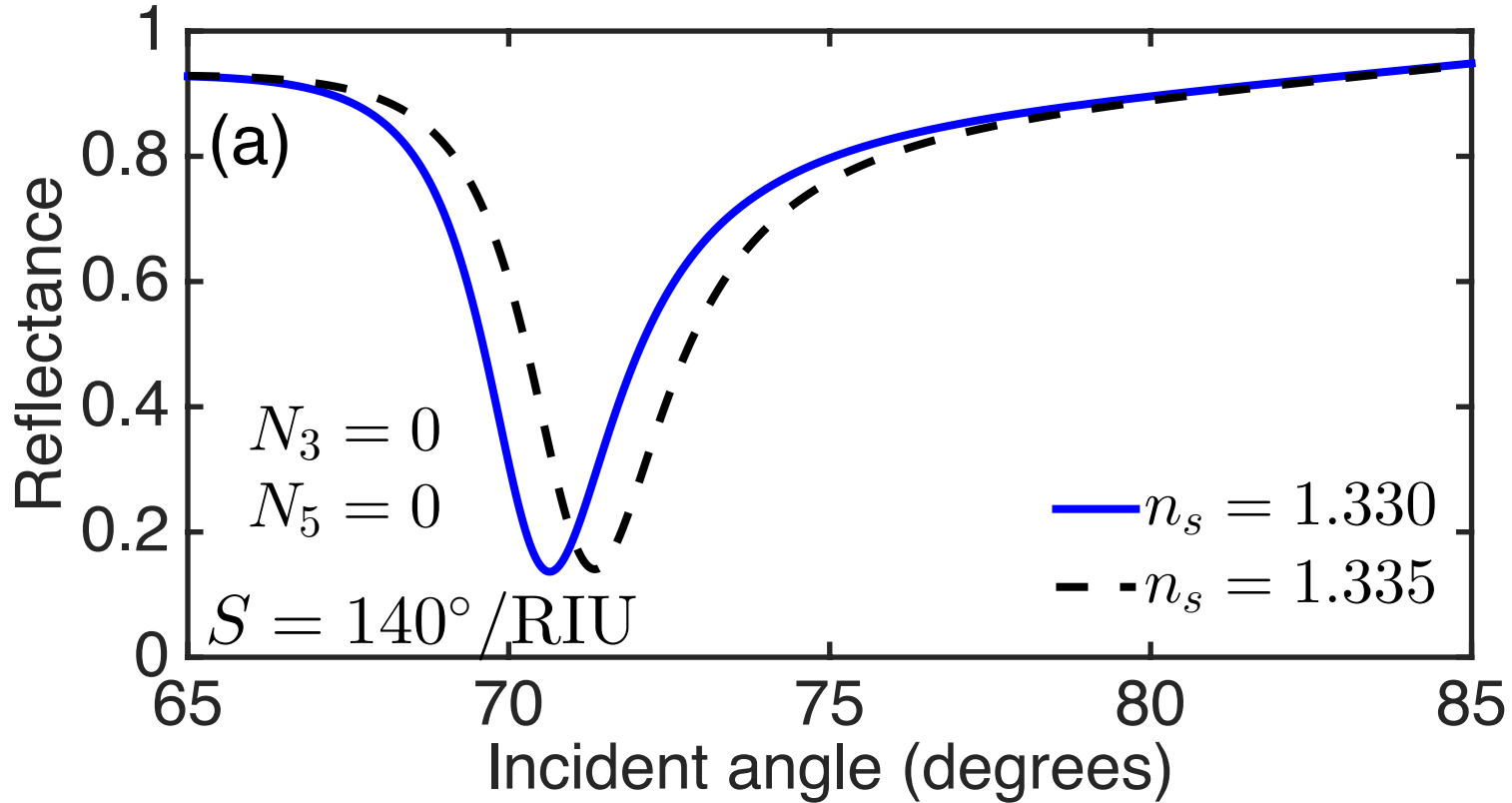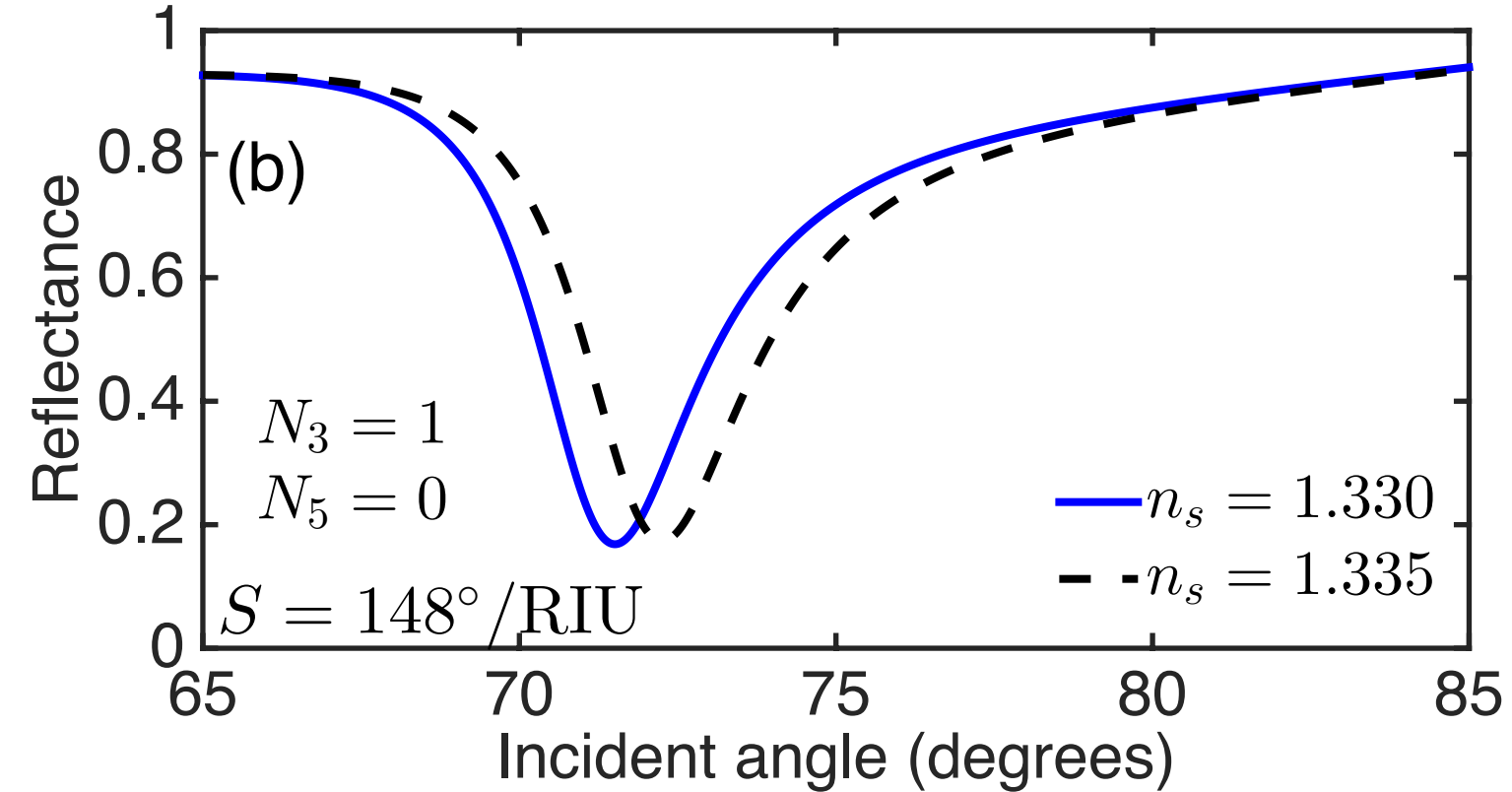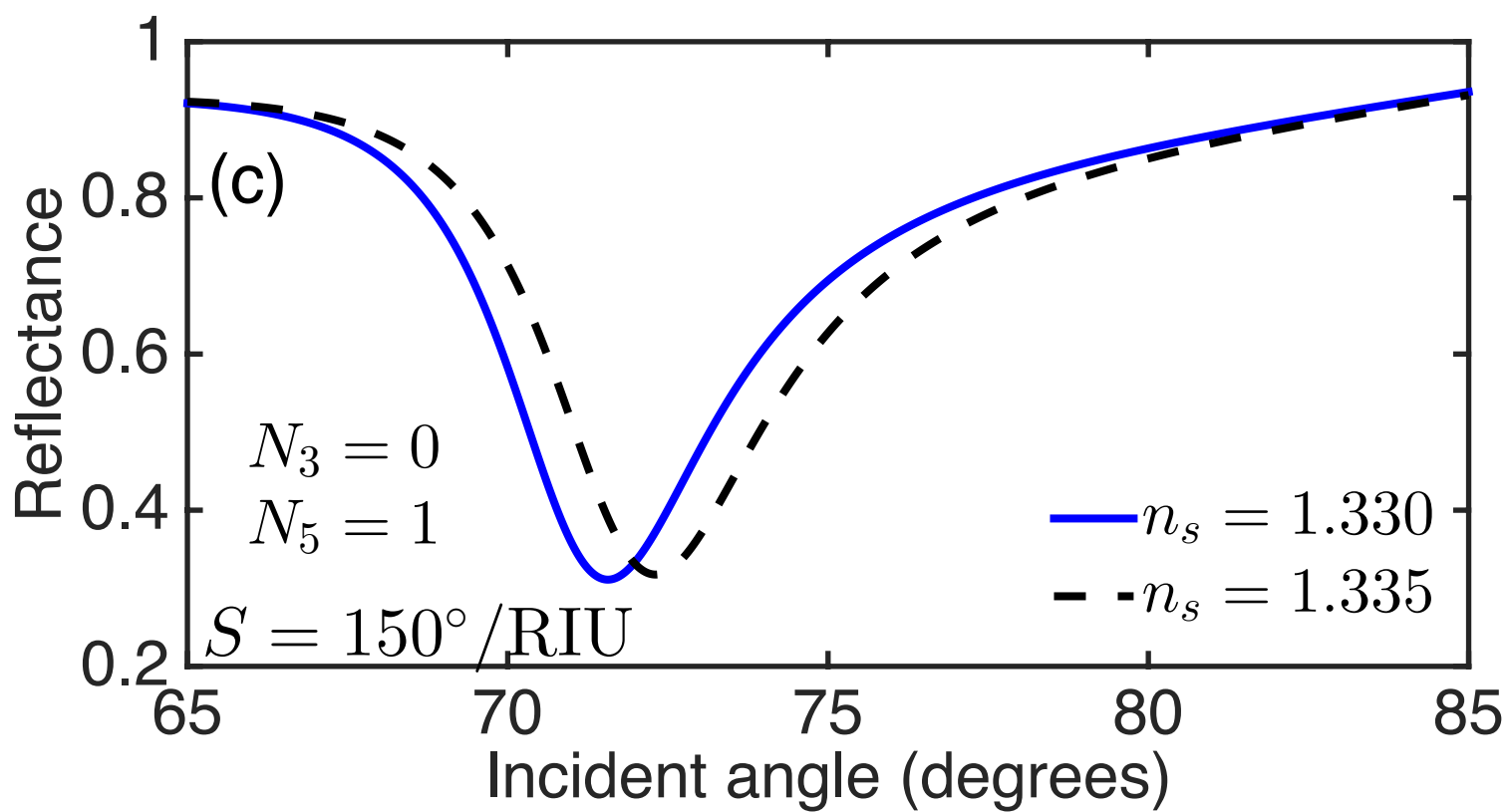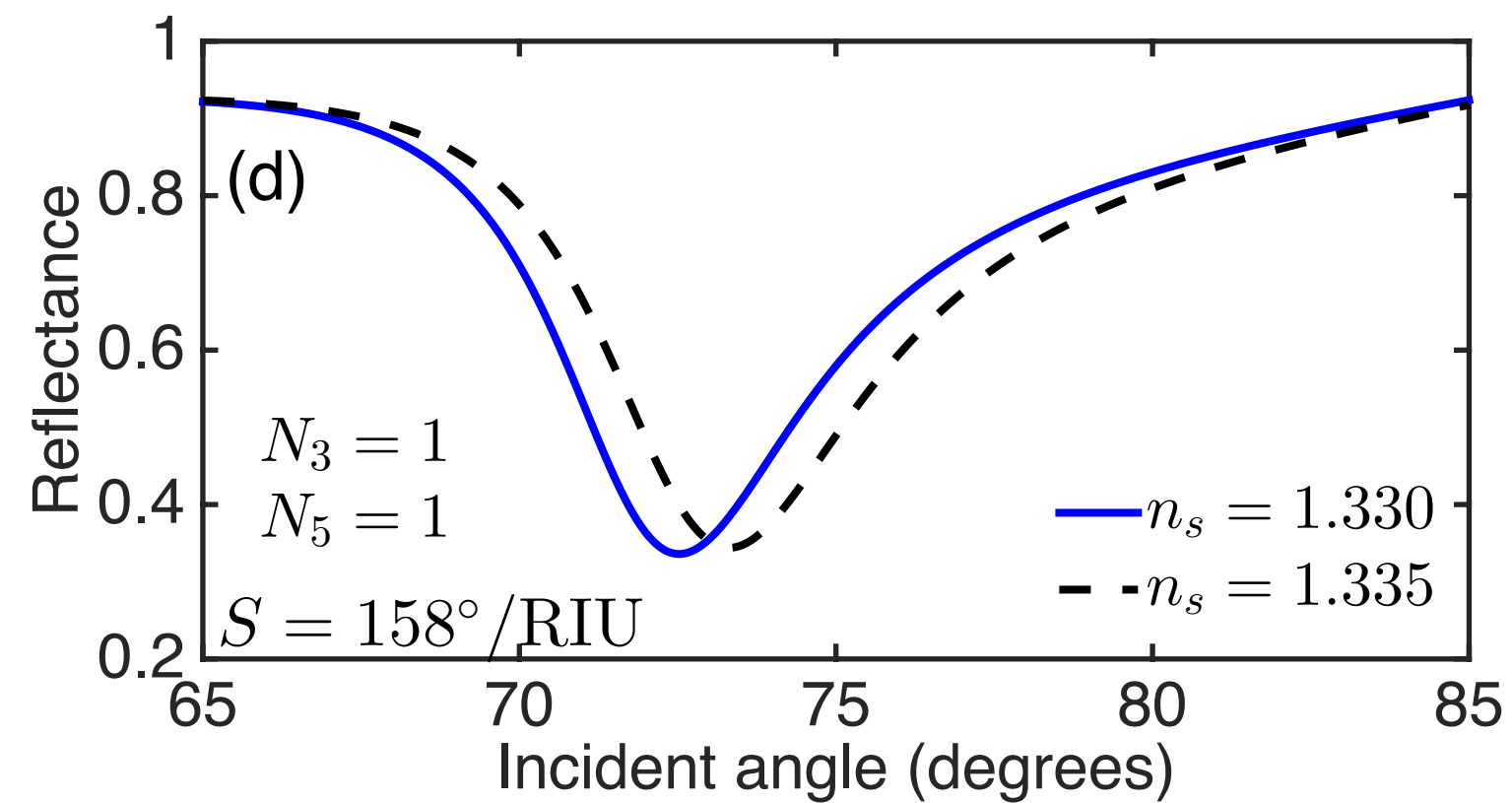

Supplement: Supplementary file 1 [file nanomaterials-09-00165-s001.zip › supporting_information_revised/figs2.pdf]

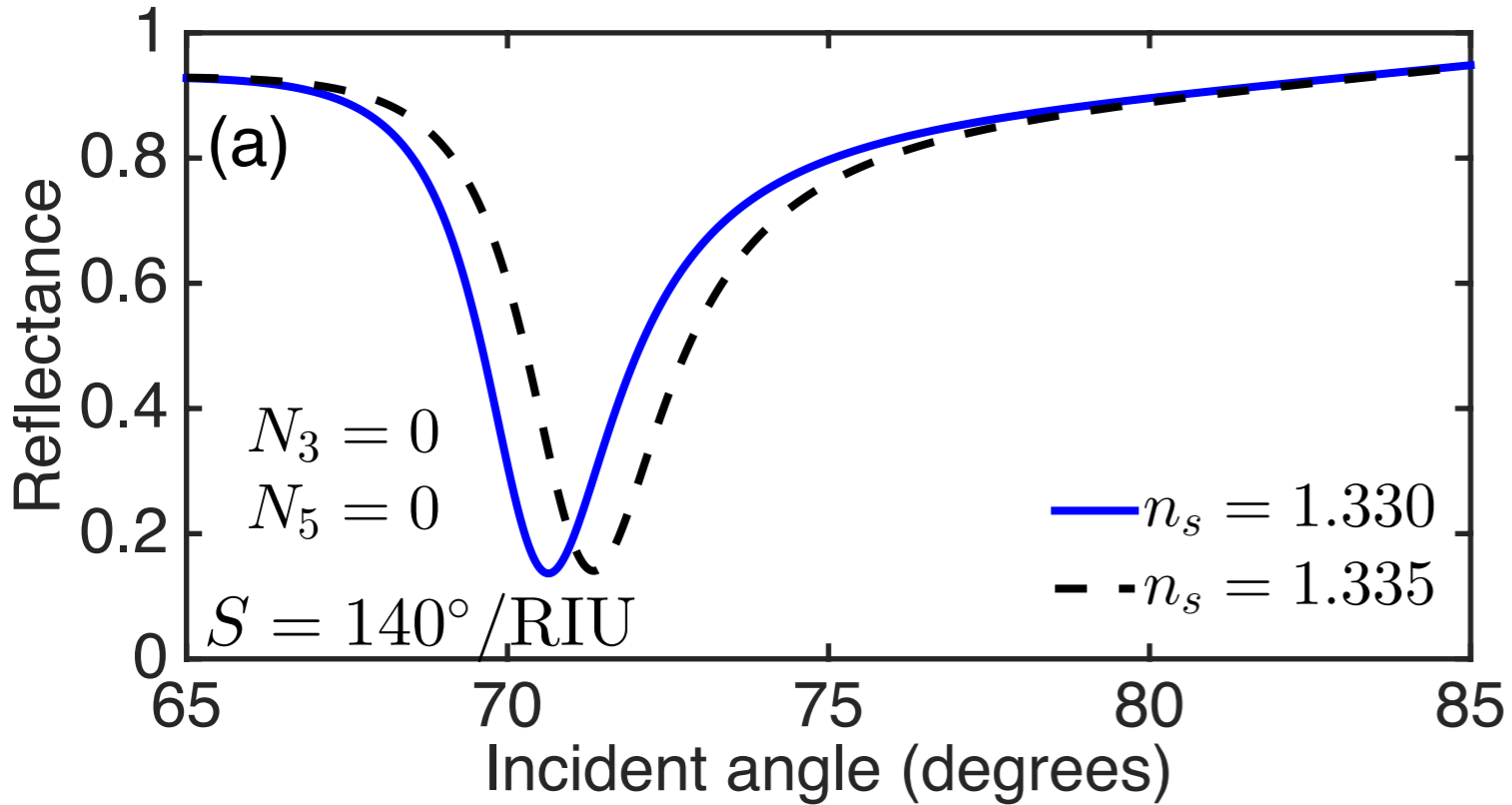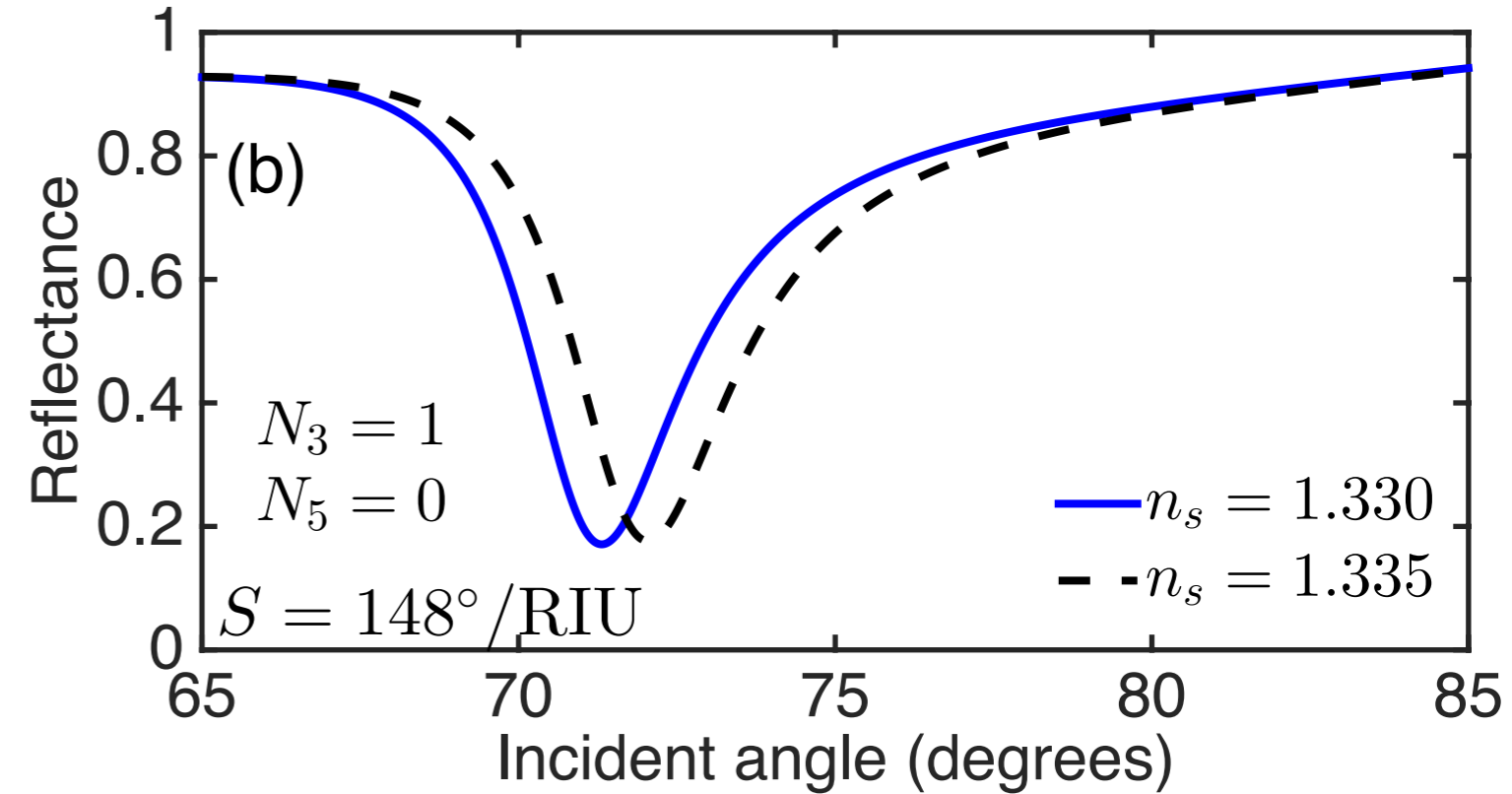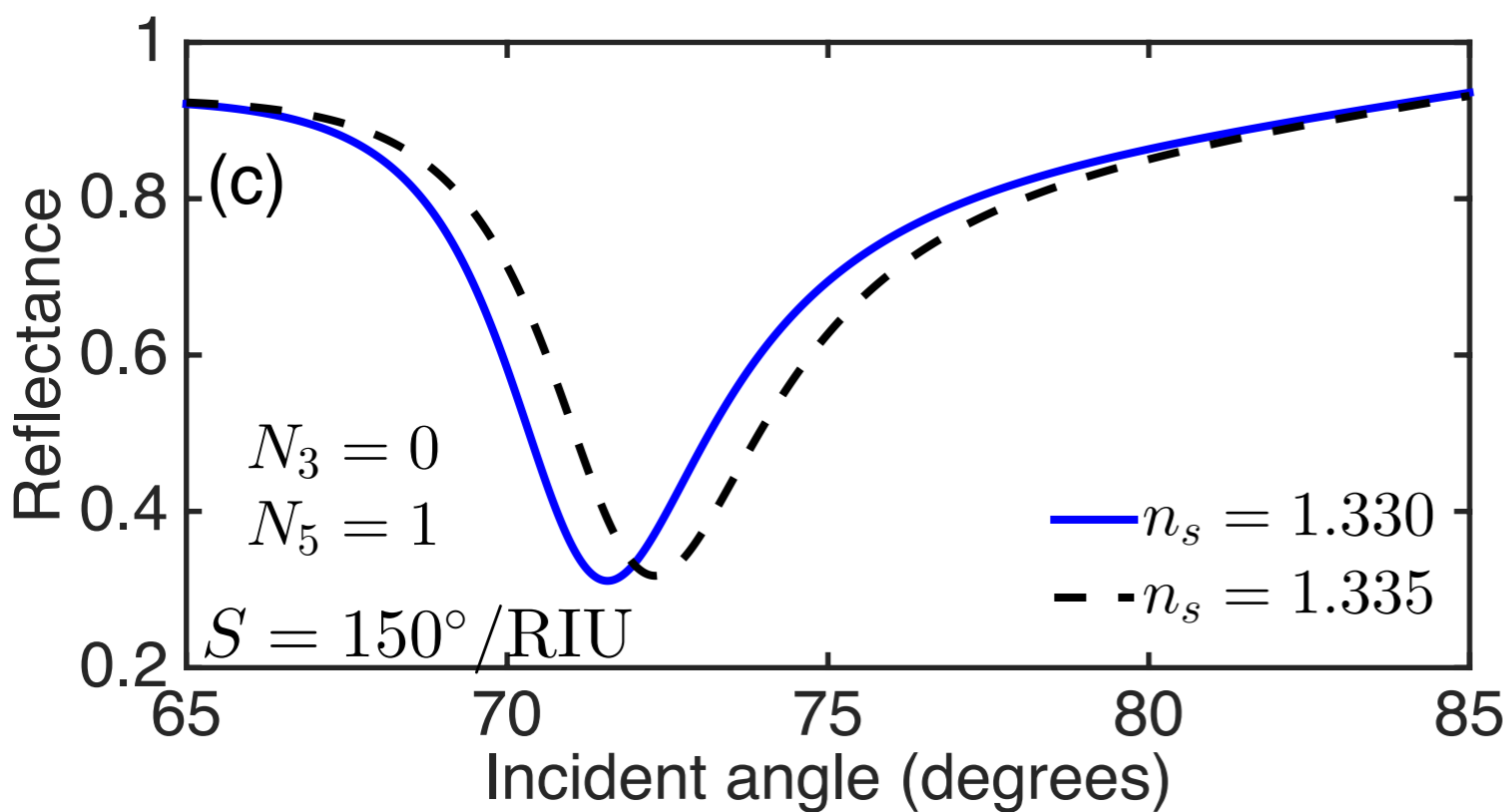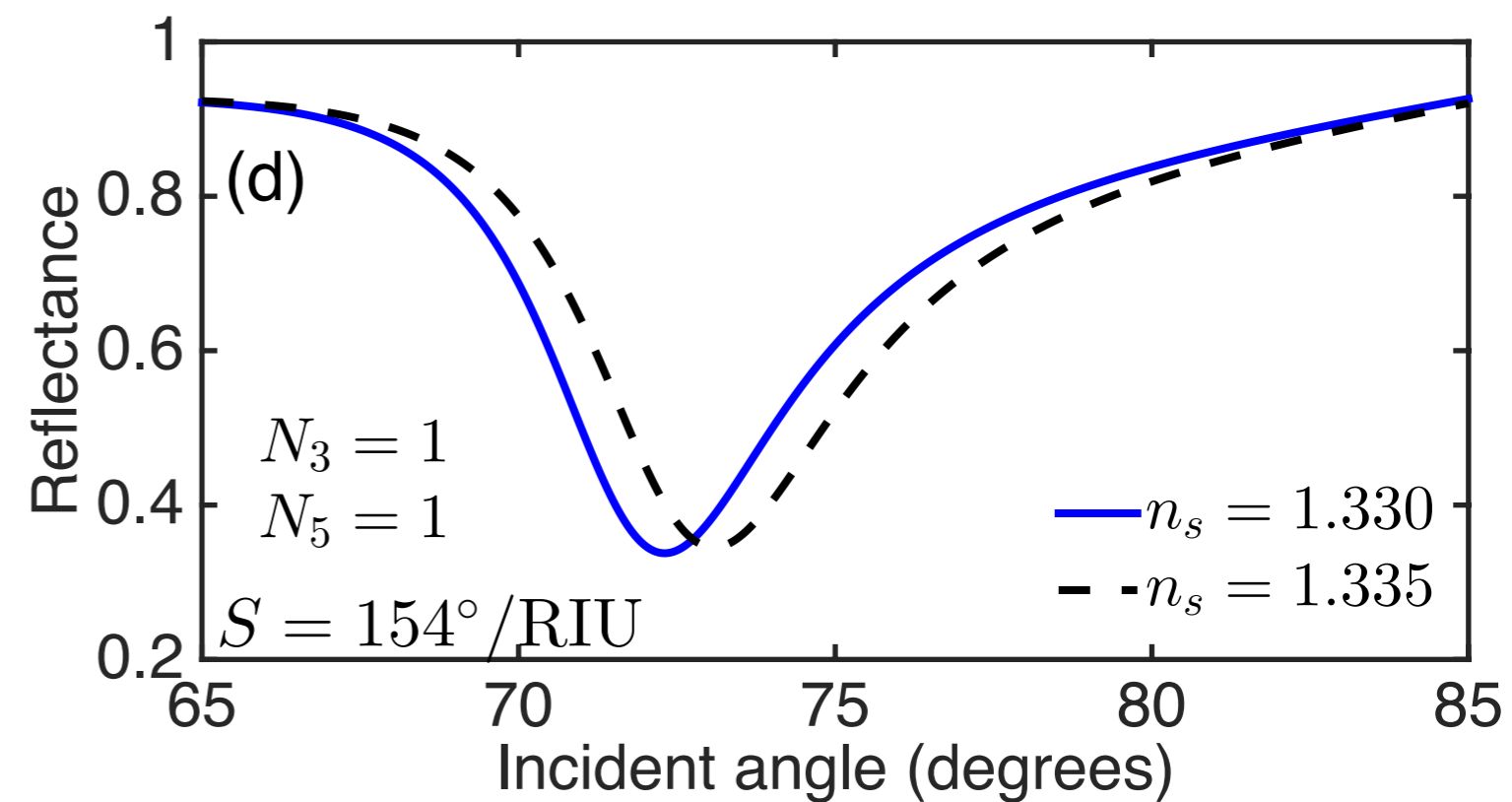

Supplement: Supplementary file 1 [file nanomaterials-09-00165-s001.zip › supporting_information_revised/figs3.pdf]

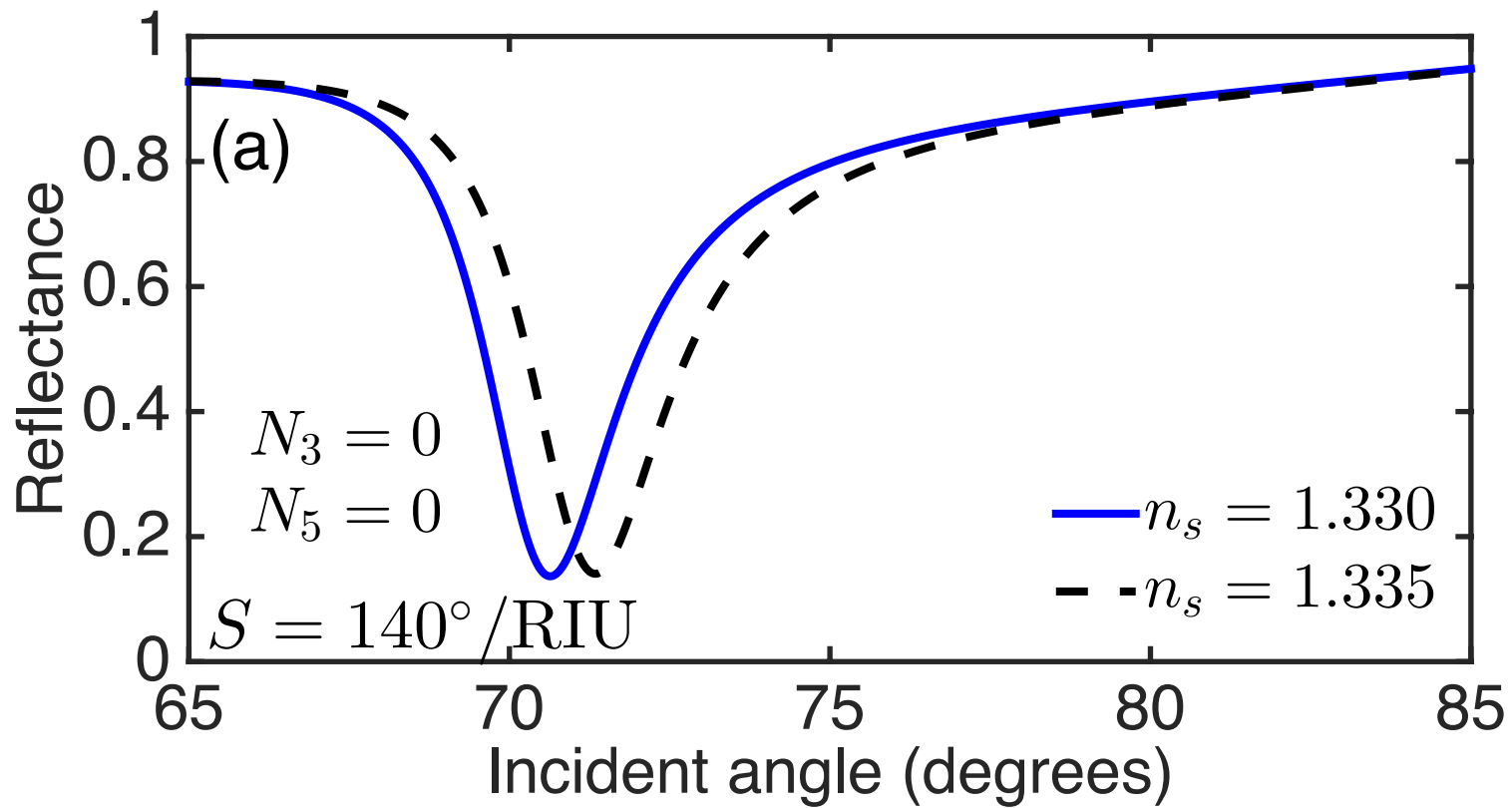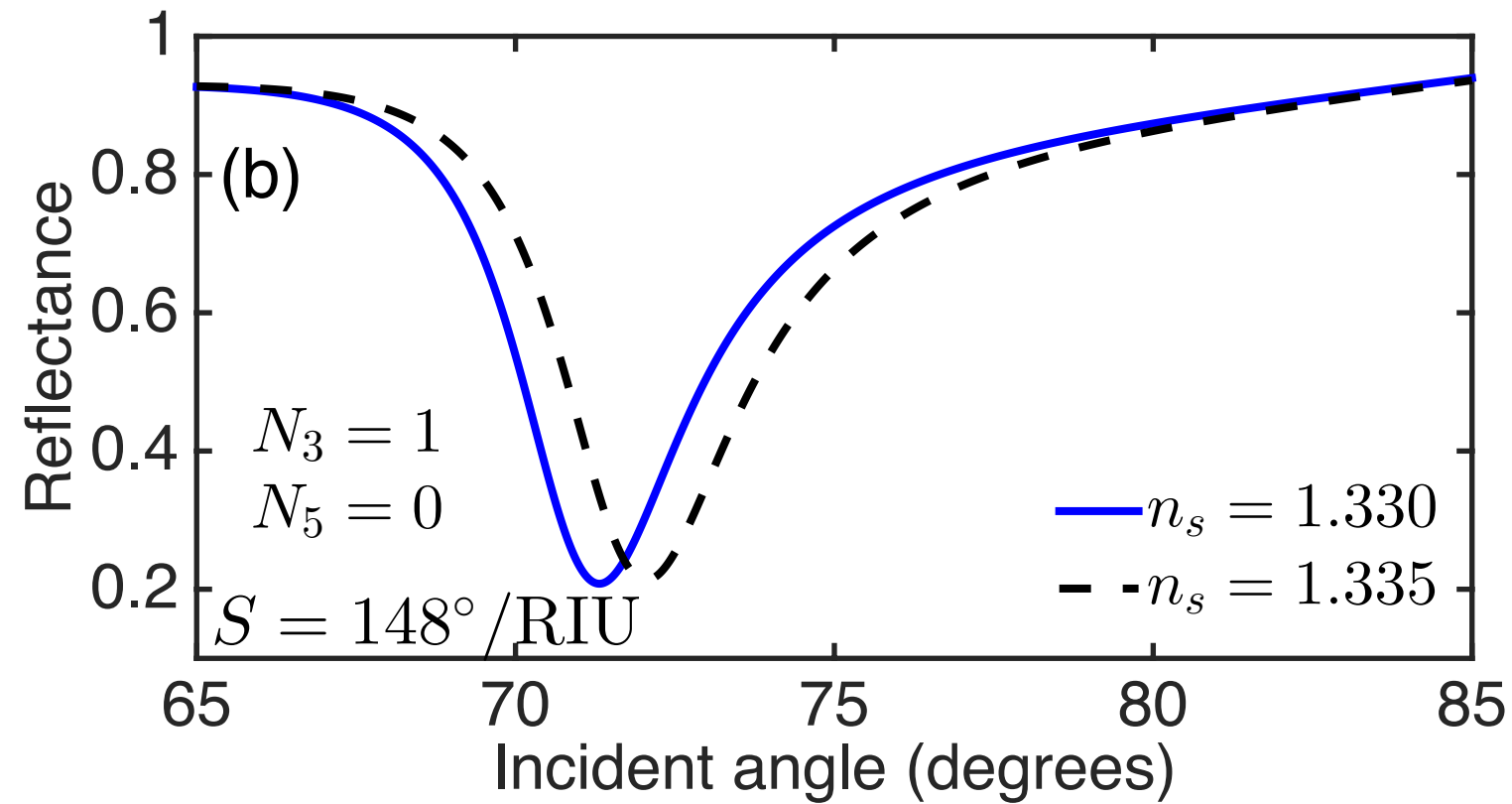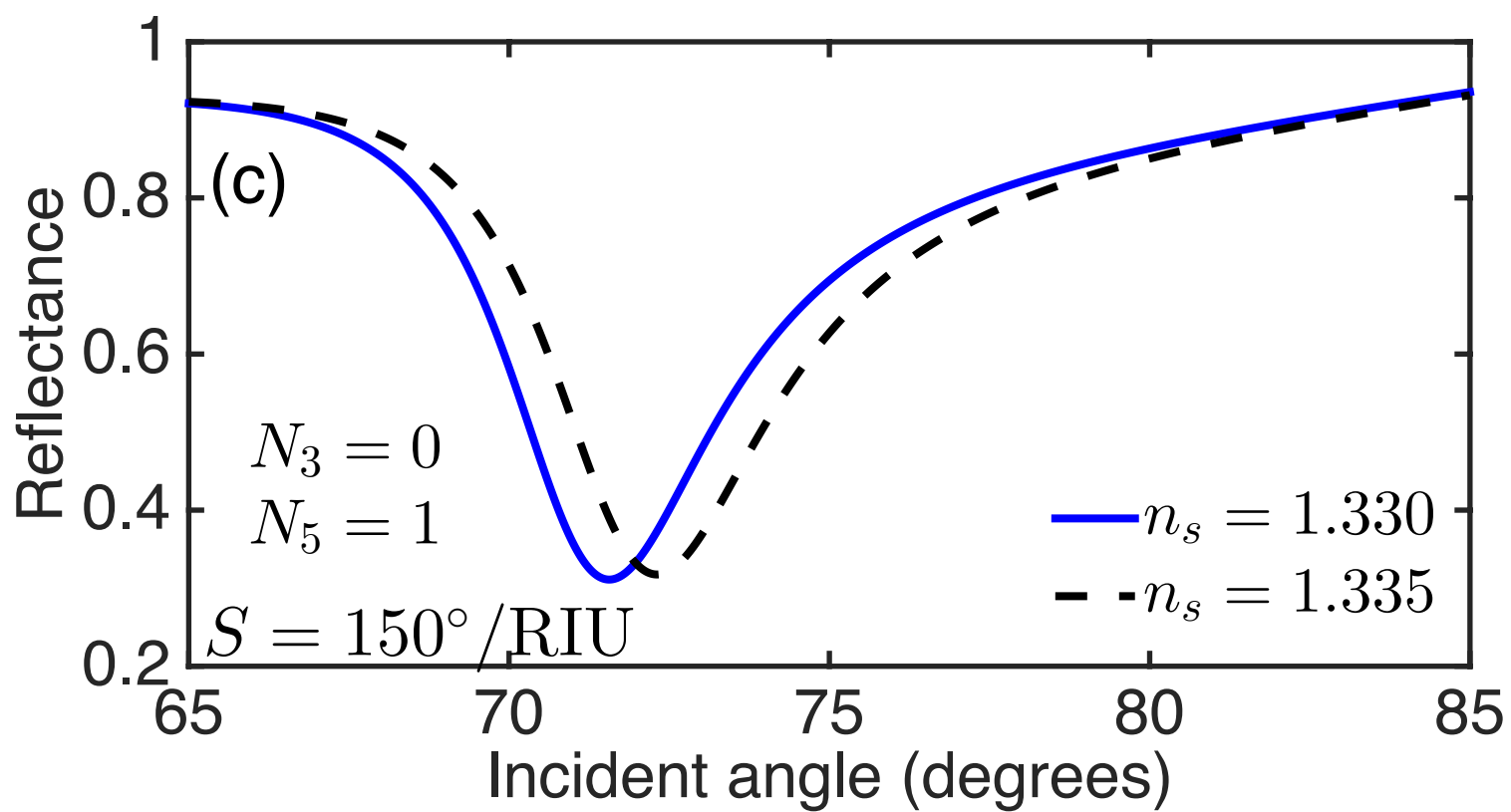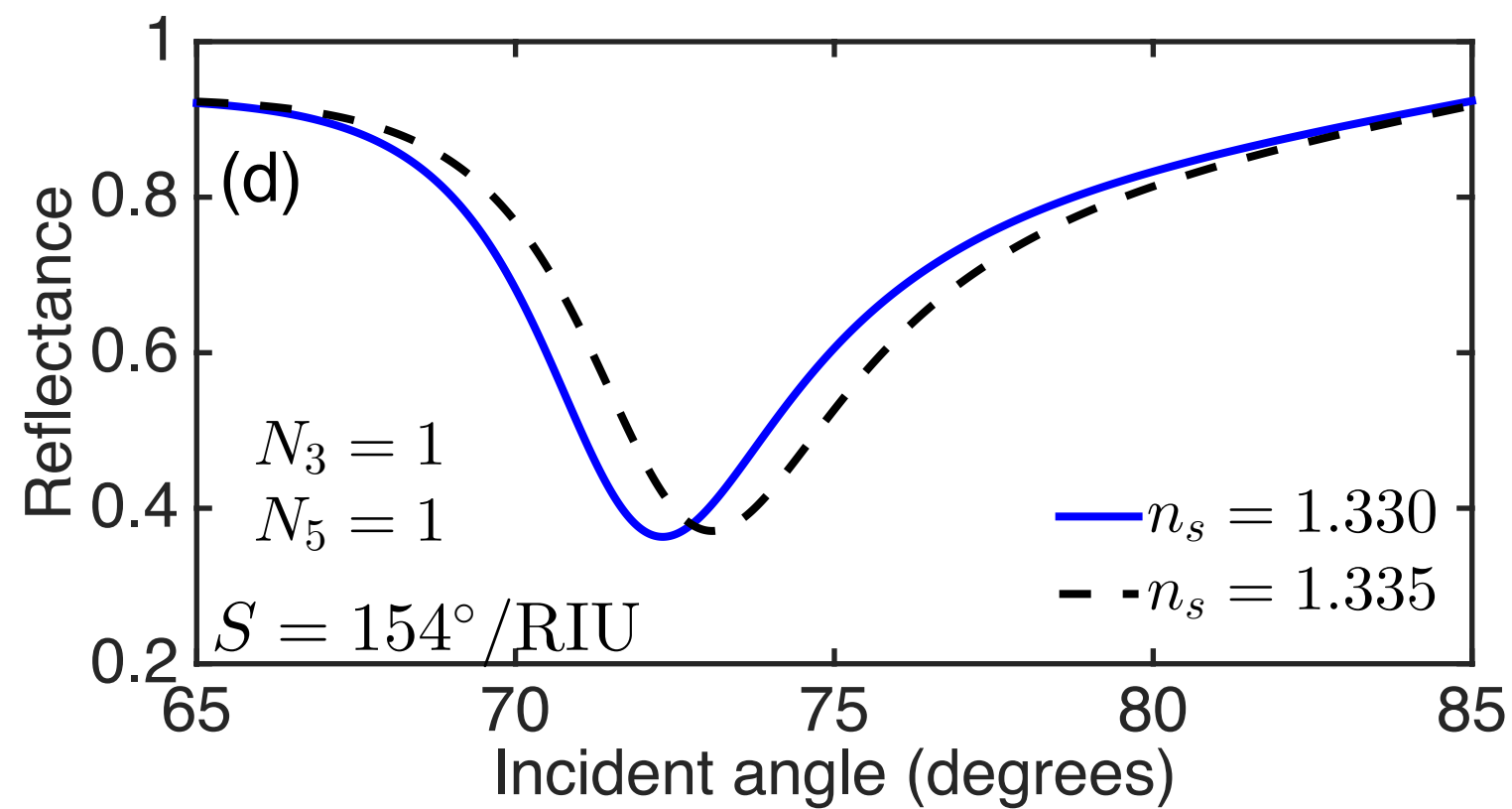

Supplement: Supplementary file 1 [file nanomaterials-09-00165-s001.zip › supporting_information_revised/figs1.pdf]
